# Editorial: Cyclic Nucleotide Phosphodiesterases (PDEs) in Immune Regulation and Inflammation

**DOI:** 10.3389/fphar.2022.950480

**Published:** 2022-07-06

**Authors:** Stefan Brocke, Paul M. Epstein, Robert Nelson, Tim Vanmierlo

**Affiliations:** ^1^ Department of Immunology, UConn Health, Farmington, CT, United States; ^2^ Department of Cell Biology, UConn Health, Farmington, CT, United States; ^3^ MindImmune Inc and University of Rhode Island, Kingston, RI, United States; ^4^ Hasselt University, Hasselt, Belgium; ^5^ Maastricht University, Maastricht, Netherlands

**Keywords:** 3′,5′-cyclic nucleotide phosphodiesterase, cell signaling, therapeutic targets, small molecule inhibitor, inflammation

Cyclic nucleotides are involved in many cellular functions and well-established regulator of immune responses and inflammation. Leukocytes play a critical role in inflammation and its modulation. Function and regulation of different subpopulations of T cells, B cells and NK cells, as well as myeloid cells, such as neutrophils, monocytes, macrophages, and dendritic cells involve activation of the cAMP pathway. Additionally, interactions between leukocytes and endothelial cells are critical during inflammatory lesion formation and can be regulated by cAMP/cGMP signaling. Cyclic nucleotides are degraded by cyclic nucleotide phosphodiesterase (PDE) enzymes. PDEs are the only enzymes known to hydrolyze cAMP/cGMP and thereby maintain spatial and temporal control over pools of cAMP/cGMP within distinct cellular compartments. PDEs are divided into 11 different gene families based on their specificity for cAMP or cGMP, structural similarity and mode of regulation. While PDEs have been recognized early as potential drug targets for anti-inflammatory drugs, bringing specific PDE inhibitors into clinical use has faced decades of challenges, mostly due to dose-limiting side effects. Major progress has been made with the approval and clinical use of select PDE4 inhibitors to treat major human inflammatory diseases. PDE4-selective inhibitors are used for oral treatment of chronic obstructive pulmonary disease, psoriatic arthritis and plaque psoriasis as well as a topical treatment for atopic dermatitis. Recent advances demonstrate a role for inflammation involved in many neurodegenerative pathologies. Due to the unique roles of specific PDE isoforms to control distinct pools of cyclic nucleotides in leukocytes, additional PDEs became a focus to study potentially novel therapeutics for inflammatory disorders.

First, basic molecular functions of PDEs cell regulation are addressed. Kurelic et al. show that PDE2A is upregulated during activation of effector/conventional CD4^+^ T cells *in vitro* Kurelic et al. Combining selective inhibitors studies, approaches to induce accumulation of cGMP through various activators and elegant real time FRET imaging, they demonstrate an increase of cAMP in non-activated and a decrease of cAMP in activated effector/conventional CD4^+^ T cells, presumably dependent on the expression of PDE2A. These studies could explain context dependent regulation of effector/conventional T cell activation and function and provide the basis for developing new therapeutic approaches to T cell mediated autoimmune disorders. Chinn et al. identify PDE4B as the primary PDE expressed in dendritic cells (DCs) and demonstrate using DC-specific depletion of Gαs that dynamic, bidirectional regulation of PDE4B expression acts as a key homeostatic regulator of cAMP levels in DCs Chinn et al. They further show that inhibition of PDE4B in Gαs-depleted DCs decreased Th2 cell differentiation, which in concert with the known phenotype of DC-selective Gαs depletion in mice suggests PDE4B inhibition as a target for Th2-allergic asthma. Golshiri et al. highlighted the complexities of exploring PDE function in disease settings, in this case the role of PDE1 in a mouse knockout model of smooth muscle cell (SMC) aging Golshiri et al. They found contrasting effects of the PDE1 inhibitor lenrispodun on different *in vivo* and *ex vivo* measures of accelerated SMC aging dependent on acute vs. chronic exposure, *in vivo* or *ex vivo* administration*,* or which pathological outcomes were being measured. Reminiscent of Chinn et al., their data suggested that inhibition of PDE1C leading to elevated cAMP/cGMP might be met by a compensatory upregulation of PDE1A that abrogates some of the lenrispodun-mediated effects. Thus a deeper exploration of these complementary pharmacologies is called for.

An area of intense research is covered by the contributions by Hoffman and Hermann et al. describing screening and testing of novel PDE inhibitors. A unique and novel approach to identify PDE inhibitors is presented by Hoffman. Hoffman developed a platform for expressing cloned PDEs in the fission yeast *Schizosaccharomyces pombe*, which allows for inexpensive, but robust screening for small molecule inhibitors that are cell permeable. An advantage of this screening method is that it is readily accessible to academic laboratories and does not require the purification of large quantities of target protein. In employing this novel approach to using yeast as a model organism for screens of PDE inhibitors as an alternative approach to enzyme-based screens, Hoffman demonstrates its significant potential both for the discovery and profiling of PDE inhibitors to treat inflammation and for inhibitors of targets such as pathogen PDEs to treat infections by parasitic nematodes. Hermann et al. explore the therapeutic utility of novel PDE4 inhibitors in treating idiopathic pulmonary fibrosis Herrmann et al. They present data on BI 1015550 as a candidate PDE4 inhibitor that has advanced into clinical trial for this indication. Their report appears to present the first published dissemination of preclinical data on this novel inhibitor. Testing oral application of BI 1015550 in two established mouse models of lung fibrosis, the authors directly measured clinically relevant endpoints of pulmonary function. Importantly, the study directly addresses the established limitations of using PDE4 inhibitors in humans by demonstrating that BI 1015550 shows preferential inhibitory activity against the PDE4B isoform. This selectivity may be of advantage in reducing the nausea/emesis side effects that are known to occur with the therapeutic use of PDE4 inhibitors.

Due to the therapeutic efficiency of PDE inhibitors in several human diseases, major efforts are spent on broadening the scope of treatments by testing inhibitors in many preclinical disease models. The emerging role of PDEs at the intersection between CNS disorders and neuroinflammation has been investigated by Pilarzyk et al. The mRNA expression of PDE11A in brain, represented solely by the PDE11A4 subtype, is restricted to the hippocampus. This group previously showed that social isolation changes subsequent social behaviors in adult mice by reducing the expression of PDE11A4 in the membrane fraction of the ventral hippocampus. In this paper they extend these findings to show that both acute and chronic social isolation decrease PDE11A4 in adult but not adolescent mice, and moreover that isolation-induced decreases in membrane PDE114A correlate with increased expression of interleukin-6 and activation of microglia. Hence these findings suggests that alteration in PDE11A4 expression may be a key molecular mechanism by which social isolation increases neuroinflammation and alters social behavior. In the context of experimental periodontitis, Kuo et al. investigated the molecular and cellular actions of a unique xanthine and piperazine derivative (KMUP-1) Kuo et al. KMUP-1 exhibits profound inhibition on PDE3, 4, and 5 and therefore increases cellular levels of cAMP and cGMP. Using *in vitro* models for inflammation, Kuo et al. showed that KMUP-1 displayed anti-osteoclastogenic and anti-inflammatory activities in a macrophage cell line mainly through a cGMP/PKG-dependent pathway. The consequent reduction of pro-inflammatory signaling pathways and cytokine expression and downregulation of crucial regulators of osteoclasts, explain the KMUP-1-induced protective functions on osteoclast maturation and alveolar bone loss in two rat models.

Collectively, the articles of this Research Topic showcase recent investigations of PDE functions in inflammation and provides an overview of their potential as targets for established and novel inhibitors for the treatment of inflammation in animal models and human disease.

